# Heat-Related Mortality and Adaptation to Heat in the United States

**DOI:** 10.1289/ehp.1307392

**Published:** 2014-04-29

**Authors:** Jennifer F. Bobb, Roger D. Peng, Michelle L. Bell, Francesca Dominici

**Affiliations:** 1 Department of Biostatistics, Harvard School of Public Health, Boston, Massachusetts, USA; 2 Department of Biostatistics, Johns Hopkins Bloomberg School of Public Health, Baltimore, Maryland, USA; 3 School of Forestry and Environmental Studies, Yale University, New Haven, Connecticut, USA

## Abstract

Background: In a changing climate, increasing temperatures are anticipated to have profound health impacts. These impacts could be mitigated if individuals and communities adapt to changing exposures; however, little is known about the extent to which the population may be adapting.

Objective: We investigated the hypothesis that if adaptation is occurring, then heat-related mortality would be decreasing over time.

Methods: We used a national database of daily weather, air pollution, and age-stratified mortality rates for 105 U.S. cities (covering 106 million people) during the summers of 1987–2005. Time-varying coefficient regression models and Bayesian hierarchical models were used to estimate city-specific, regional, and national temporal trends in heat-related mortality and to identify factors that might explain variation across cities.

Results: On average across cities, the number of deaths (per 1,000 deaths) attributable to each 10°F increase in same-day temperature decreased from 51 [95% posterior interval (PI): 42, 61] in 1987 to 19 (95% PI: 12, 27) in 2005. This decline was largest among those ≥ 75 years of age, in northern regions, and in cities with cooler climates. Although central air conditioning (AC) prevalence has increased, we did not find statistically significant evidence of larger temporal declines among cities with larger increases in AC prevalence.

Conclusions: The population has become more resilient to heat over time. Yet even with this increased resilience, substantial risks of heat-related mortality remain. Based on 2005 estimates, an increase in average temperatures by 5°F (central climate projection) would lead to an additional 1,907 deaths per summer across all cities.

Citation: Bobb JF, Peng RD, Bell ML, Dominici F. 2014. Heat-related mortality and adaptation to heat in the United States. Environ Health Perspect 122:811–816; http://dx.doi.org/10.1289/ehp.1307392

## Introduction

Heat exposure has been associated with adverse health outcomes, including higher rates of all-cause and cardiovascular mortality and emergency hospitalizations, across a range of study designs, and covering geographical regions worldwide (Basu 2009; Hajat et al. 2006; Michelozzi et al. 2009; Semenza et al. 1996; Ye et al. 2012). In the United States, extreme heat accounts for the largest cause of mortality due to severe weather, followed by hurricanes and tornadoes [National Weather Service (NWS) 2013]. Under climate change, higher average temperatures, increased summertime temperature variability, and more extreme heat events are anticipated to greatly exacerbate the health impacts of heat [Li et al. 2013; Intergovernmental Panel on Climate Change (IPCC) 2007b; Meehl and Tebaldi 200; Zanobetti et al. 2012]. However, several factors, referred to as adaptations, have the potential to reduce the extent of the impacts. Factors that may modify the population’s vulnerability to heat over time include both intrinsic biological factors, such as life stage and health status, and extrinsic factors encompassing environmental, social, and health system conditions (IPCC 2007a).

A major source of evidence on the mortality impacts of heat comes from multisite time-series studies (Anderson and Bell 2009; Basu 2009; Braga et al. 2002; Curriero et al. 2002; O’Neill et al. 2005). These studies typically estimate community-specific and national average acute heat-related mortality risks, defined as the association between day-to-day changes in summer temperature (heat exposure) and day-to-day changes in mortality, adjusted for other potential confounding factors (e.g., air pollution) that vary from day to day. These studies generally assume that heat-related mortality risk remains constant over the whole study period of several years.

Far fewer studies have investigated how heat-related mortality might be changing slowly over time in the United States (Barnett 2007; Barrecca et al. 2012; Davis et al. 2003a, 2003b; Guo et al. 2012; Sheridan et al. 2009). These studies have provided some evidence that heat-related mortality risk has declined, but considerable uncertainty remains as to whether the decrease is continuing in more recent years and whether heat remains significantly associated with mortality in the present. Moreover, whether the rates of temporal change in heat-related mortality differ across communities and population subgroups and the particular factors that explain why different communities might exhibit different rates of temporal change have not been identified.

We hypothesized that the strength of the association between acute heat exposure and mortality risk is continuing to change slowly over time due to adaptation, change in vulnerability, and susceptibility. We also hypothesized that the rate of this temporal change would vary both by individual-level factors (age, cause of death) and community-level factors (geographical region, climate). Finally, we hypothesized that cities with larger increases in air conditioning (AC) prevalence over time would have larger temporal declines in heat-related mortality risk. To gather evidence addressing these hypotheses, we extended the standard approach for analyzing multisite time-series data (Dominici et al. 2000, 2007; Samet et al. 2000a) in order to allow the location-specific and national average heat-related mortality risks to vary smoothly over time, and we applied this methodology to estimate city-specific, regional, and national temporal trends in heat-related mortality risk in the United States using the most recently available national data on daily mortality, weather, and air pollution.

## Methods

*Data*. Time-series data covering 106 million people in 105 large U.S. urban communities (see Supplemental Material, Figure S1) were obtained as part of the National Morbidity and Mortality Air Pollution Study (NMMAPS) (Samet et al. 2000b). This database, which has been updated with data from 1987 to 2005, consists of parallel time series of daily weather variables from the National Climatic Data Center (2013), daily air pollution concentrations from the U.S. Environmental Protection Agency’s (EPA) Air Quality System (U.S. EPA 2013), and counts of the number of daily deaths from the National Center for Health Statistics. Mortality counts exclude deaths due to external causes (e.g., accidents, suicides, homicides) and are stratified by age category (< 65, 65–74, and ≥ 75 years) and cause (cardiovascular, respiratory, other). A full description of NMMAPS, along with the derivation of geographical regions, has been previously reported (Dominici et al. 2002; Samet et al. 2000b). Data were restricted to the summer months: June, July, and August.

AC prevalence data from alternate years during the period 1989–2005, obtained from the U.S. Census Bureau’s American Housing Survey (AHS), were available for 79 of the 105 NMMAPS cities (U.S. Census Bureau 2013). The AHS is a longitudinal survey of housing characteristics, collected from a representative sample of housing units by the U.S. Census Bureau, and results are publicly available (U.S. Census Bureau 2013). We estimated the change in AC prevalence over time as the slopes obtained from regressing AC prevalence on year separately within each city.

*Statistical analysis*. To estimate temporal changes in the relative risk of short-term mortality associated with elevated summer temperatures, we used the two-stage methodology previously developed for NMMAPS (Dominici et al. 2000, 2002). At the first stage, we specified a Poisson regression model of the number of daily deaths *Y_itg_* for age group *g* on day *t* in city *i*:

log *E*(*Y_itg_*) = γ*_i_*_0_ + β*_i_*(*t*)*x_it_* + γ*_i_*_1_*dow_t_* + γ*_i_*_2_*agecat_g_* + *ns*(*t*, 2 df/3 months; γ*_i_*_3_), [1]

where *x_it_* is the average daily temperature, *dow_t_* is a categorical variable for day of the week, *agecat_g_* is a categorical variable for age group (comparing 65–74 and ≥ 75 to < 65 year age groups), and *ns*(·) denotes natural cubic splines with the specified number of degrees of freedom (df) and knots at quantiles. The smooth function of calendar time *ns*(*t*, 2 df/3 months; γ*_i_*_3_) accounts for within-summer seasonality and long-term trends (Anderson and Bell 2010; Bobb et al. 2011; Zanobetti and Schwartz 2008); thus, the model accounts for slowly changing factors such as demographic changes to estimate acute mortality risk associated with the same day’s heat exposure, β*_i_*(*t*). We allowed each year to have a different coefficient on daily temperature *x_it_*, but constrained the change in the coefficient over time to be linear by modeling β*_i_*(*t*) as

β*_i_*(*t*) = β*_i_*_0_ + β*_i_*_1_(year*_t_* – 1987). [2]

Combining models 1 and 2 yields a within-city model with an interaction term between temperature and year. Here β*_i_*_0_ is the city-specific log relative risk of mortality associated with a 1°F increase in the same day’s summer temperature in 1987 (the first year of the study period), and β*_i_*_1_ is the yearly change in the log relative risk. The number of excess deaths (per 1,000 deaths) attributable to each 10°F increase in temperature on summer day *t* was calculated as 1,000{exp[10β*_i_*(t)]–1}.

In the second stage, we combined the city-level estimates for β*_i_* = (β*_i_*_0_, β*_i_*_1_) across locations to estimate the national average, time-varying risk of heat-related mortality using Bayesian hierarchical models (Dominici et al. 2000). We assumed

β̂*_i_* |β*_i_* ~ *N*(β*_i_*, V̂*_i_*); independent, *i* = 1,…, *I* [3]

β*_i_* ~ *N*(β*, Σ), [4]

where β̂*_i_* are the estimated coefficients for the *i*th city, β*_i_* are the true city-level coefficients, V̂*_i_* is the estimated variance of β̂*_i_*, β* are the national average coefficients, and Σ is the across-cities variance of the true city-specific coefficients. Noninformative priors were placed on β* and Σ, and an independent sampling procedure was used for posterior inference (Everson and Morris 2000).

To estimate separate temporal trends by age group and cause of death, model 1 was expanded to include interaction terms of each variable with indicator variables for age group and mortality subtype. Regional estimates of temporal changes were obtained by expanding model 4 to include indicator variables for each U.S. region. We investigated potential effect modification by average temperature by expanding model 4 to include the city-level average of daily temperatures over the study period, and effect modification by central AC prevalence by expanding model 4 to include the city-level change in prevalence over the study period.

Parameter estimates from the Bayesian hierarchical models (i.e., of the regional and national temporal trends, of the differences in temporal trends across age groups and regions, and of effect modification), were deemed “statistically significant” if 95% posterior credible intervals (PI) excluded zero. We conducted extensive sensitivity analyses for the model specification, which are described in the Supplemental Material.

## Results

**Figure 1:**
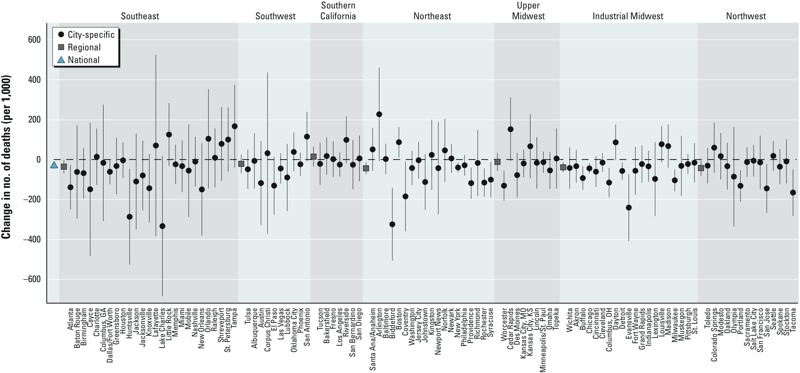
Estimates of the change, from 1987 to 2005, in the excess number of deaths (per 1,000 deaths) attributable to each 10°F increase in the same day’s summer temperature. Intervals correspond to 95% confidence intervals (city-specific estimates) or PIs (regional and national estimates).

Figure 1 shows the change, from 1987 to 2005, in heat-related mortality risk, defined as the number of excess deaths, per 1,000 deaths, attributable to each 10°F increase in the same day’s summer temperature. Nationally, the number of excess heat-related deaths (per 1,000 deaths) declined from 51 (95% PI: 42, 61) in 1987 to 19 (95% PI: 12, 27) in 2005, for a total reduction in risk of 32 (95% PI: 18, 45) deaths, per 1,000 deaths (left-most point of Figure 1). Heat-related mortality risk decreased over the study period in 74 of the 105 cities (corresponding to the negative point estimates of change in risks in Figure 1), and for 18 of these cities the temporal decline was statistically significant. The national average temporal trend remained negative and statistically significant across different specifications of the statistical model, with larger temporal declines in the estimated risk based on a nonlinear temperature–mortality exposure–response function and under larger temperature lags (see Supplemental Material, Figure S2A). Allowing for a more flexible time-varying coefficient model (i.e., relaxing the linearity assumption) suggested that the rate of temporal change in the national trend may be slowing for years past 2000 (see Supplemental Material, Figure S2B).

**Figure 2:**
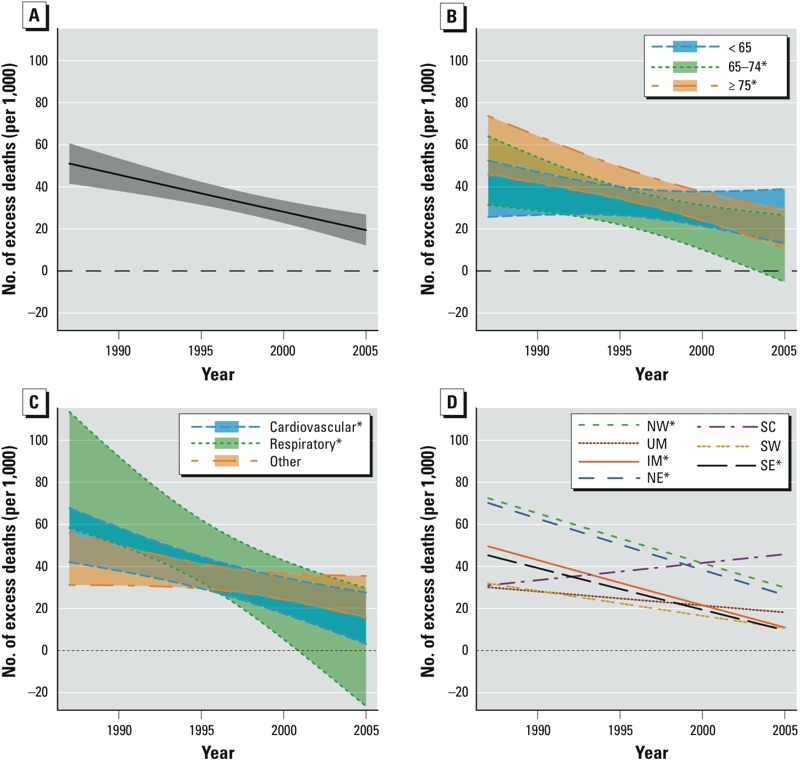
Temporal trends, from 1987 to 2005, in the excess number of deaths (per 1,000 deaths) attributable to each 10°F increase in the same day’s summer temperature, nationally in the United States, on average across age groups (*A*), and stratified by age group (*B*), cause of death (*C*), and geographical region (*D*). Abbreviations: IM, Industrial Midwest; NE, Northeast; NW, Northwest; UM, Upper Midwest; SC, Southern California; SE, Southeast; SW, Southwest. Asterisks in the key denote statistically significant trends.

We next sought to identify whether the temporal trends varied by age group and cause of death (Figure 2B,C; see also corresponding numerical results in Supplemental Material, Table S1). Nationally, heat-related mortality risk declined for each age group, with the 65–74 and ≥ 75 year groups exhibiting a similar change over time (the oldest group had higher baseline risk in 1987), and the < 65-years age group exhibiting only a small, nonstatistically significant, decrease over time. Although the heat-related mortality risk for the ≥ 75-years age group was greater than for the < 65-years age group at the beginning of the study period, by 2005 they had converged to similar levels. Heat-related mortality risk declined for each cause of death, and the decline was statistically significant for cardiovascular and respiratory deaths. The largest rate of decline occurred for respiratory mortality, although respiratory deaths contribute less to total mortality (of the approximately 13.5 million deaths covered by our study, 8.7% were due to respiratory causes compared with 42.7% from cardiovascular causes).

Because the risk of heat-related mortality varies across geographical regions (Anderson and Bell 2009; Medina-Ramon and Schwartz 2007), we posited that the temporal trends would reflect similar heterogeneity. Across regions, risks declined most in the Northeast, Northwest, and Industrial Midwest, followed by the Southeast (Figure 2D; see also Supplemental Material, Table S1). For one region (Southern California) we estimated an increase in risk over the study period, but the estimate was not statistically significant.

**Figure 3:**
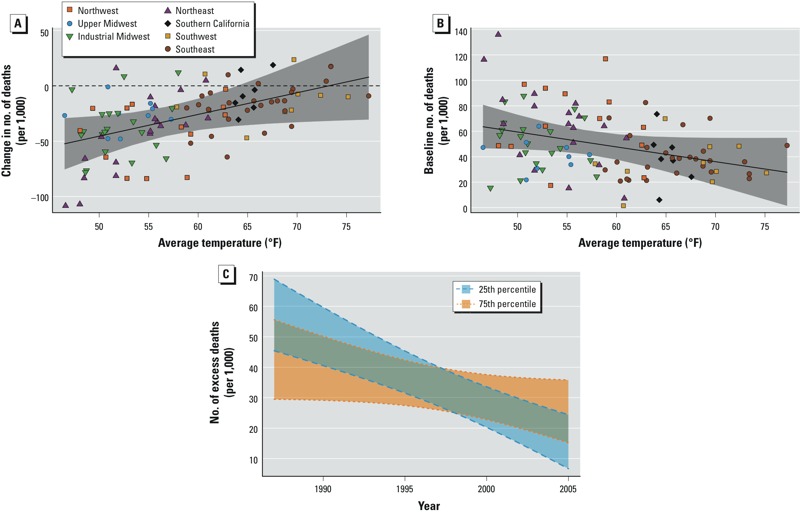
Effect modification by local climate. City-specific (posterior mean) estimates of the change, from 1987 to 2005, in heat-related mortality risk (*A*) and baseline (1987) heat-related mortality risk (*B*), plotted against the average temperature over the study period. (*C*) Temporal trends, from 1987 to 2005, in heat-related mortality risk for cities at the 25th percentile (52.0°F) and 75th percentile (64.5°F) of average temperature. Heat-related mortality risk is defined as the excess number of deaths (per 1,000 deaths) attributable to each 10°F increase in the same day’s summer temperature. The solid lines show the estimated associations, and the shaded bands denote 95% PIs.

Even with the decline in risk over time, heat remained associated with excess mortality through 2005 in all regions, and the association was statistically significant in the Northeast, Northwest, and Southern California regions. Allowing for separate regional trends by age group, we found that among the four regions with statistically significant temporal trends, the temporal decline in risk was driven by the decline among the ≥ 75-years age group (see Supplemental Material, Table S1). Next, having found that the largest declines occurred mostly in the northern regions, we investigated whether climate in particular modified the change in heat-related mortality risk over time. We found statistically significant effect modification, with cities with cooler climates having larger temporal declines in risk than cities with warmer climates, although these cities also generally had larger risks of heat-related mortality at the beginning of the study period (Figure 3; see also Supplemental Material, Table S2).

**Figure 4:**
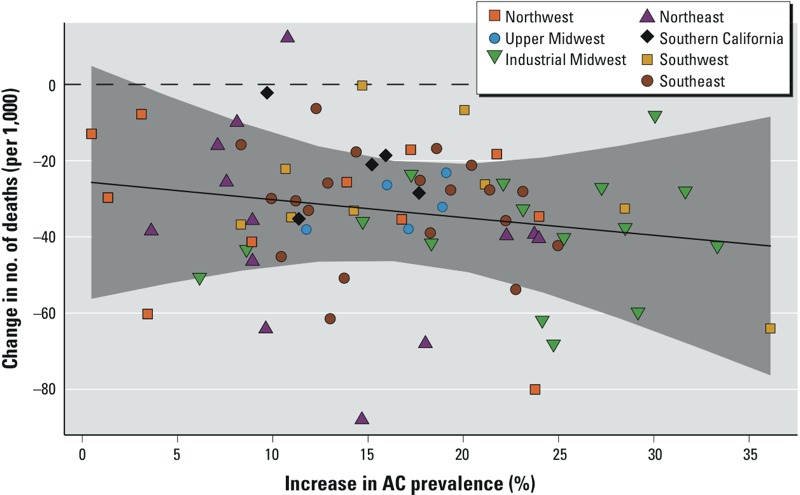
Effect modification by central AC prevalence. City-specific (posterior mean) estimates of the change, from 1987 to 2005, in the excess number of deaths (per 1,000 deaths) attributable to each 10°F increase in the same day’s summer temperature, plotted against the change in central AC prevalence over that period. The solid line shows the estimated associations, and the shaded band indicates 95% PIs.

One factor that has been proposed as a major contributor to reductions in heat-related mortality over time is AC use (Barnett 2007; Davis et al. 2003a, 2003b; Sheridan et al. 2009). Over our study period, central AC prevalence, defined as the percentage of homes with central AC, increased in all 79 cities with available AC data (see Supplemental Material, Figure S3). On average across cities, central AC prevalence was 48% at the beginning of the study period and increased by approximately 1% each year. Cities with larger increases in central AC prevalence over the study period tended to have slightly larger reductions in heat-related mortality risk over time, but the association was not statistically significant (Figure 4; see also Supplemental Material, Table S2). We found similar and nonstatistically significant effect modification when we considered a broader definition of AC prevalence that also included homes with window units, and also when we restricted the analysis to the 49 cities having baseline (1989) AC prevalence < 64% (which is equal to 100% minus the maximum change in AC prevalence over the study period) to account for the possibility that cities with high AC prevalence at baseline may have reached market saturation.

## Discussion

This study provides strong evidence that acute (e.g., same-day) heat-related mortality risk has declined over time in the United States, even in more recent years. This evidence complements findings from U.S. studies using earlier data from the 1960s through the mid-1990s on community-specific mortality rates (Davis et al. 2003a, 2003b), as well as European studies that found temporal declines in heat-related mortality risk (Carson et al. 2006; Donaldson et al. 2003; Kysely and Plavcova 2012; Schifano et al. 2012), and supports the hypothesis that the population is continually adapting to heat.

Although the mortality impact of heat has declined, we found that statistically significant risks of heat-related mortality remain through 2005. Moreover, with the increase in average summertime temperature, and the greater frequency, duration, and intensity of extreme heat events due to climate change, even these lower mortality risks attributable to heat will likely continue to contribute to a large burden of adverse health effects across the population. For example, based on the most recent (2005) estimates of heat-related mortality risk, an increase in average daily temperature by 5°F [a central value across U.S. climate projections (Karl et al. 2009)] would lead to an additional 1,907 deaths per summer across all cities in our study population (see Supplemental Material, Table S3). This would exceed the total number of severe weather fatalities that occurred across the United States in 2012 by more than a factor of three (NWS 2013). Thus, even with the recent adaptation trends, there is continued potential for further gains in mitigating excess heat-related deaths in a changing climate.

To gain insight into the potential for differential adaptation by location and population subgroup, we investigated variation in the temporal trends in heat-related mortality risk across age categories and geographical regions. Rates of decline differed by age group, with the temporal trends both nationally and regionally being driven by declining risks among the older (≥ 75 years of age) population. Northern regions exhibited the largest declines in risk, and, in particular, cities with cooler climates tended to have larger temporal declines in heat-related mortality risk than cities with hotter climates. However, cities with cooler climates also exhibited higher risks at the beginning of the study period, suggesting that these communities may be “catching up” to communities in hotter climates that had already adapted to some extent before our study period.

To gain insight into the mechanisms of adaptation, we investigated whether the temporal decline in heat-related mortality risk could be attributed to the increasing prevalence of central AC over time. We focused on AC as a potential adaptation, because this factor has been widely hypothesized as a major contributor to reducing the adverse health impacts of heat (Barnett 2007; Davis et al. 2003a; Sheridan et al. 2009). We evaluated the degree to which variation across cities in the temporal trends in heat-related mortality risk could be explained by increases in central AC prevalence, finding that declining risks from 1987 to 2005 could not be solely attributed to expanded AC prevalence during that period. One possible explanation is that, because the cost of running AC on high heat days can be prohibitive for some households (Sheridan 2007), higher AC prevalence does not automatically imply higher usage. Also, without AC data for population subgroups, we could not directly evaluate whether groups that are more susceptible to heat (e.g., the elderly) may have experienced different trends in AC prevalence.

Disentangling the set of factors that have contributed to declining heat-related mortality is challenging in part because of limited data availability (e.g., lack of AC usage data or even AC prevalence data for all years and cities) and in part because many potential adaptive factors are changing slowly over time and lack sufficient year-to-year variability. Cardiovascular deaths comprise a substantial portion of heat-related mortality (Basu 2009), and incidence of myocardial infarction and subsequent cardiovascular mortality has decreased in recent decades (Ford and Capewell 2011; Yeh et al. 2012). Consequently, the decline in risk of heat-related mortality may reflect the general decline in risk factors (e.g., smoking, cholesterol levels) and well-established improvements for the treatment of cardiovascular disease that have driven declines in adverse cardiovascular outcomes (Ford and Capewell 2011). Interventions specific to reduce short-term heat exposure may have also played a role. For example, extensive heat–health warning systems and public health response programs have been implemented in several U.S. cities, including Philadelphia, Pennsylvania, (introduced in 1995) and Phoenix, Arizona, (in 2002) (U.S. EPA 2006). These programs often contain specific measures targeted toward the elderly population, which could be one reason why heat-related mortality declined most rapidly for the oldest age group. The reduction in heat-related mortality could also reflect a shift from mortality to morbidity, whereby some individuals are admitted to the hospital rather than dying from heat exposure. Other possible factors include changes in activity patterns (e.g., less time spent outside), changes in the built environment (e.g., more green spaces), physiological adaptations, and greater awareness of the dangers of extreme heat.

Our findings have important implications for studies assessing the future health impacts of heat in a changing climate. Projections of future heat-related mortality rely on numerous factors that exhibit a wide range of uncertainty, including future greenhouse gas emissions, global and regional climate models, population growth, statistical models for estimating the health effects, the degree to which baseline levels of morbidity and mortality will change over time, and the degree to which individuals and communities will adapt to changes in the distribution of heat (Bobb et al. 2011; Huang et al. 2011; IPCC 2007b; Peng et al. 2011). Most studies have used central estimates of the risks of heat averaged over an historical study period as the basis for future projections, which implicitly assumes no change in risk over time (Huang et al. 2011). By failing to account for the temporal decline in risk, these studies may overestimate mortality impacts of future heat, especially if the observed trends continue. Furthermore, if health impacts studies ignore differences across age groups in the temporal trends, they may not accurately characterize future vulnerability to heat, especially given anticipated changes in the population’s age structure over the course of the century. The age-group–specific estimates of the changes in risk over time presented here may be combined with projections of future population demographics in order to more accurately project future heat-related mortality under climate change.

This work also has implications for developing targeted interventions aimed at strengthening the population’s capacity to adapt to heat. We found that mortality risks in the eldest (≥ 75 years) age group differed from the youngest (< 65 years) at baseline but were statistically indistinguishable by the end of the study period. This suggests that although the elderly have historically been more susceptible to extreme heat (Basu 2009), interventions that are more broadly targeted to reduce vulnerability to heat over the lifespan should be considered moving forward. In addition, our finding that the decline in risk of heat-related mortality from 1987 to 2005 could not be fully attributed to increased central AC prevalence over the study period suggests that a range of other factors (e.g., declining cardiovascular mortality rates) likely explain the decline. Evidence of declining heat risks in locations in northern Europe where AC prevalence remains much lower than in the United States (Carson et al. 2006; Donaldson et al. 2003), also supports the causal role of other factors. Thus, sole emphasis on expanding AC prevalence as a mechanism to reduce future heat-related mortality is likely to miss key opportunities for adapting to and mitigating the effects of heat, especially if AC has already reached market saturation in certain areas. Furthermore, more sustainable adaptations, such as passive cooling systems (Santamouris et al. 2007), may be preferred over widespread adoption of AC, with its high energy requirements and contribution to poor air quality and climate change.

Several limitations in the data should be acknowledged. First, the aggregation of mortality data into broad age categories precludes finer information on temporal trends in younger adults or children. As such, we were unable to detect whether risks among potentially vulnerable younger populations (e.g., outdoor workers, high-school athletes), may also be changing over time. In addition, because we considered data from only urban and suburban areas, our results may not be applicable to rural communities with different demographic, socioeconomic, or housing characteristics.

We focused on acute heat exposure (same-day or few-days lag), rather than on heat waves (consecutive days of high temperatures). Temporal trends in heat wave mortality risk could be estimated similarly, by specifying a time-varying coefficient on the heat wave indicator variable. Our temporal trend estimates also did not account for the potential for short-term mortality displacement, which could occur if heat exposure causes deaths that otherwise would have occurred just a few days later in the absence of the exposure (Braga et al. 2001). Evidence on the contribution of mortality displacement to estimates of heat-related mortality is mixed and appears to vary based on population characteristics and other factors (Basu 2009). Moreover, although the implication of mortality displacement on heat-related mortality risk estimates would be attenuation, its possible effects on the corresponding temporal trend estimates is less clear and warrants further study. Finally, although this study focused on heat, changing vulnerability to cold temperatures could be investigated using similar methods because climate change may also mitigate cold-related mortality (Watkiss et al. 2009).

## Conclusions

We have proposed a framework to study adaptation by leveraging information on the temporal trends in acute heat-related mortality risk to gain insight into which communities and subpopulations are adapting to heat, as well as into the mechanisms by which adaptation may be occurring. Our methodology to assess the role of expanded AC prevalence in reducing the risk of heat-related mortality could be applied to evaluate any potential adaptive strategy. Previously, the main way potential adaptive factors had been identified was to investigate whether differences across communities in a particular factor were associated with differences in heat risks (Anderson and Bell 2009; Curriero et al. 2002; Medina-Ramon and Schwartz 2007; O’Neill et al. 2005). One challenge of that approach is that numerous factors differ across communities that could confound any results. Because the approach we propose here examines how changes in potential adaptive factors over time are associated with temporal changes in the heat risks, our approach is less prone to confounding by factors that remain constant (or nearly constant) over the period of study. Future studies could apply this methodology to systematically identify the drivers of declining heat-related health impacts, thereby informing the development of targeted interventions to mitigate the effects of heat in a changing climate.

## Supplemental Material

(2.4 MB) PDF
