# Supplementary material for: Heat-Related Mortality and Adaptation to Heat in the United States
Source: Environ Health Perspect. 2014 Apr 29;122(8):811–6. doi: 10.1289/ehp.1307392 (PMC4123027; doi:10.1289/ehp.1307392)
Supplement: (2.4 MB) PDF [file ehp.1307392.s001.pdf]

Environmental Health Perspectives doi:10.1289/ehp.1307392

Supplemental Material

Heat-Related Mortality and Adaptation to Heat in the United States

Jennifer F. Bobb, Roger D. Peng, Michelle L. Bell, and Francesca Dominici

Table of Contents **Page**

Sensitivity analysis methods 2

Reference 4

Figure S1. Study locations of the 105 US urban communities 5

Figure S2. Sensitivity analysis for estimating the national temporal trend in acute heat-related mortality risk 6

Figure S3. Time trends in central air conditioning (AC) prevalence 7

Figure S4. Estimated temperature-mortality exposure-response function for the summer months (June–August) in the 20 largest cities 8

Table S1. Posterior mean estimates (95% posterior intervals) of heat-related mortality risk in 1987 and 2005 and of its temporal change from 1987 to 2005 on average across all cities ("National") and by age, region, cause of death, and age within region 9

Table S2. Effect modification by average temperature over the study period ("Local Climate") and by change in central air conditioning (AC) prevalence over the study period 11

Table S3. Average number of deaths per summer (1987 to 2005) and the excess number of heat-related deaths attributable to a 5°F increase in average daily temperature 12

Sensitivity analysis methods

Our initial first-stage, within-city regression model (equation [1] in the main text) assumed that the temperature-mortality association when restricted to the summer months was linear, which we found was a reasonable assumption for the majority of cities. Supplemental Material, Figure S4 shows the time-invariant temperature-mortality exposure-response function during the summer months (June–August) for the 20 largest cities estimated using a penalized cubic spline model, which allows the data to determine the degree of smoothing.

Departures from linearity were considered by modeling temperature using natural cu- bic splines with 3 degrees of freedom (DF) and knots at quantiles. We also considered different lags for the temperature covariate by replacing average daily temperature x_{it} with the average of current and previous days' temperatures at different lags. Alternate values for the DF of the smooth function of calendar time were considered: 10, 19, and 57 DF, which correspond to 0.5, 1, and 3 DF per 3 months time, respectively. Sensitivity to the linear varying-coefficient model (equation [2] in the main text) was assessed by modeling $\beta_i(t)$ using natural cubic splines with knots at the years 1991, 1996, and 2001.

We also fit models that included adjustment for current day's fine particulate matter ($PM_{2.5}$) and ozone. Because the pollutants are generally not measured every day, locations with fewer than 500 days with pollution data (out of 1748 total summer days) were excluded. This left 96 cities with sufficient data for the analysis adjusting for ozone and 53 cities for the analysis adjusting for $PM_{2.5}$. Note that if ozone is considered as a potential mediator of the temperature-mortality association as proposed by Reid et al. (2012), then our original model not including ozone is estimating (temporal trends in)

the total effect of heat on mortality, which includes the effect mediated by ozone, while estimates from the model adjusting for ozone are of the unmediated effect of heat on mortality, under the usual assumptions for causal inference.

Reference

Reid CE, Snowden JM, Kontgis C, Tager IB. 2012. The Role of Ambient Ozone in Epidemiologic Studies of Heat-Related Mortality. Environ Health Perspect. doi:10.1289/ehp.1205251.

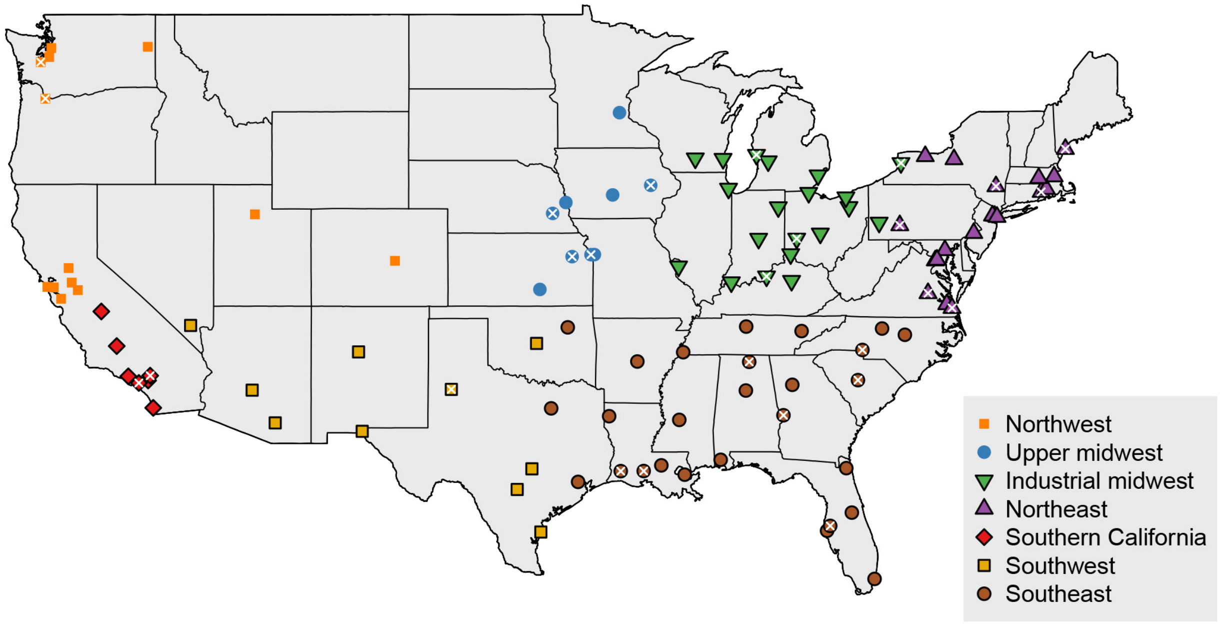

Figure S1. Study locations of the 105 US urban communities. Locations are color-coded by region. Locations with air conditioning (AC) prevalence data are represented by solid circles; locations without AC data are represented by empty squares.

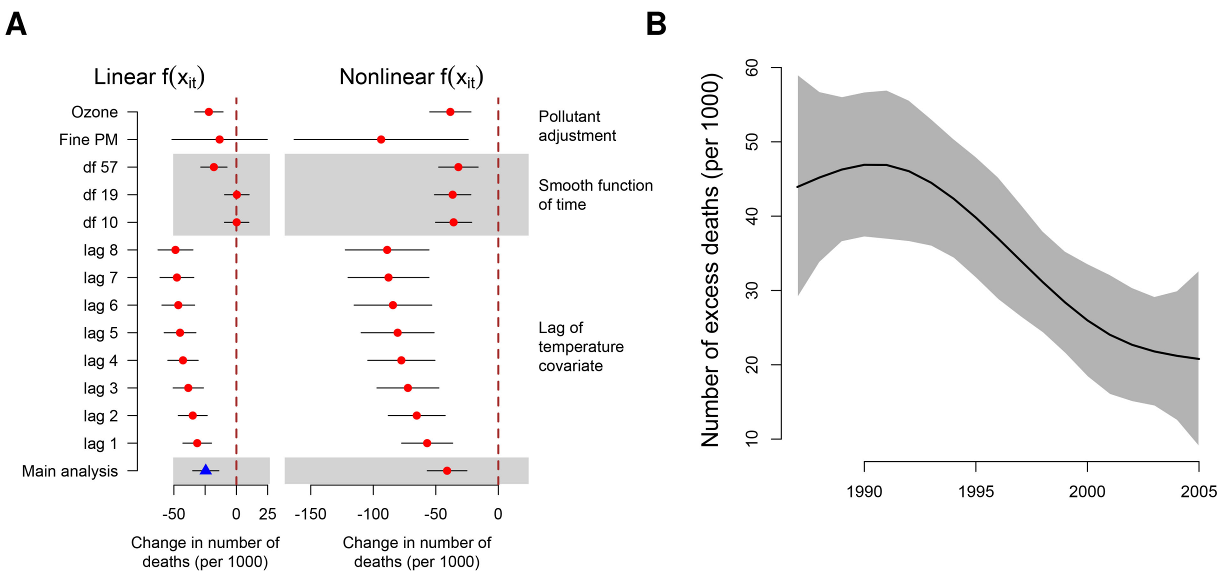

Figure S2. Sensitivity analysis for estimating the national temporal trend in acute heat-related mortality risk. **(A)** National average change, from 1987 to 2005, in the excess number of deaths (per 1000 deaths) attributable to an increase in temperature from the 50th to the 95th percentile (of daily summer temperature) for different model specifications, where the temperature-mortality exposure-response model $f(x_{it})$ is assumed to be linear (left panel) or allowed to be more flexible (right panel). "Main analysis" refers to the model with lag 0 (same day) temperature and 36 degrees of freedom (df) in the smooth function of time. The blue triangle corresponds to the results presented in Figure 1 of the main text. **(B)** National average excess number of deaths (per 1000 deaths) attributable to each 10˚F increase in the same day's summer temperature plotted over time, for the more flexible (spline model) of the time-varying heat effect. Shaded bands correspond to 95% posterior intervals.

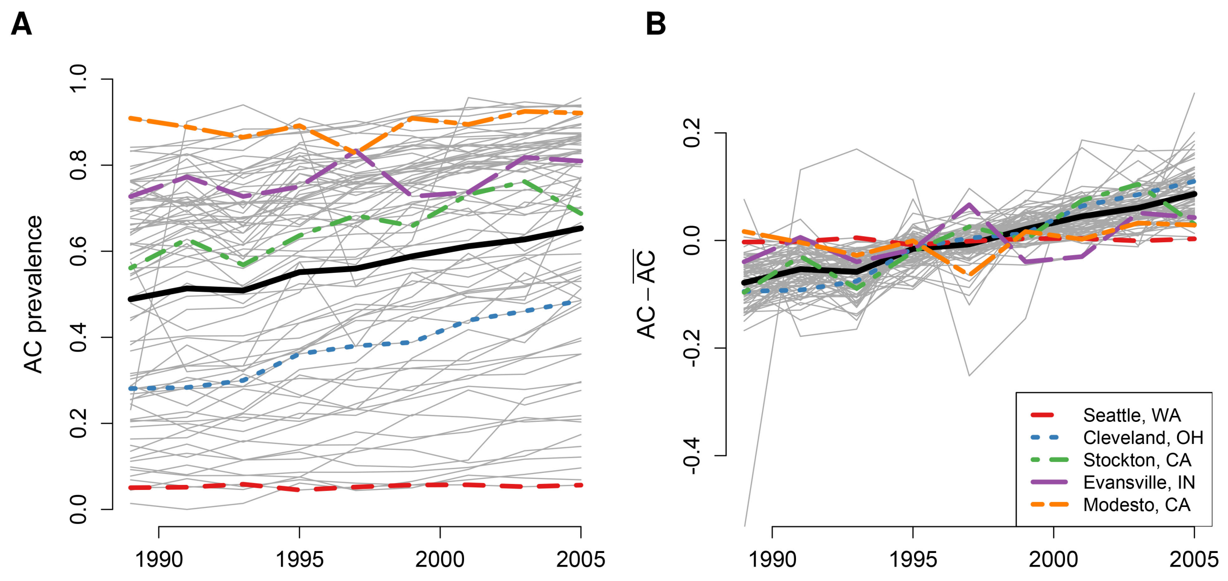

Figure S3. Time trends in central air conditioning (AC) prevalence. **(A)** Plot of AC prevalence in the 79 cities (shown in Supplemental Material, Figure S1) with available data. Data was available every two years, from 1989 to 2005. **(B)**, Same as **(A)** but each city's yearly AC prevalence has been centered by subtracting the average of that city's AC prevalence over the study period. Trends from 5 cities, representing different percentiles of average AC prevalence over the study period, are highlighted: Seattle, WA (percentile: 0%); Cleveland, OH (25%); Stockton, CA (50%); Evansville, IN (75%); and Modesto, CA (100%).

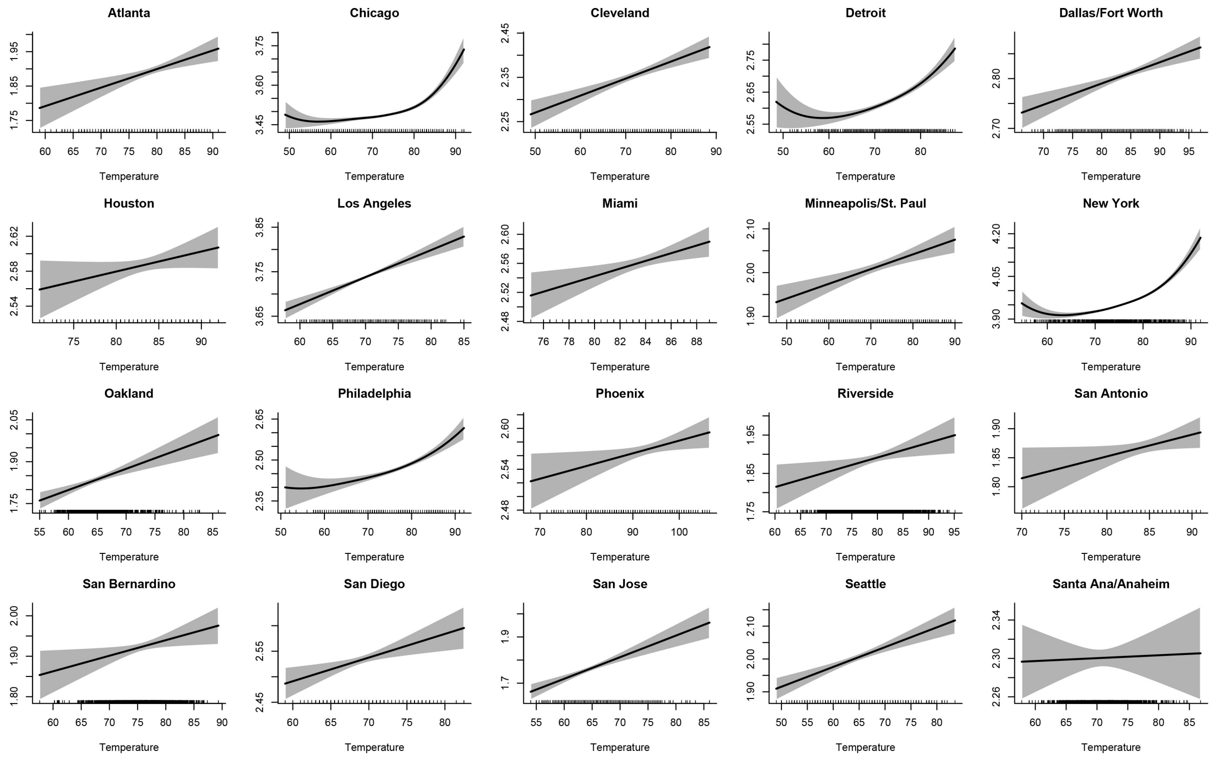

Figure S4. Estimated temperature-mortality exposure-response function for the summer months (June–August) in the 20 largest cities.

Table S1. Posterior mean estimates (95% posterior intervals) of heat-related mortality risk in 1987 and 2005 and of its temporal change from 1987 to 2005 on average across all cities ("National") and by age, region, cause of death, and age within region. Heat-related mortality risk is defined as the excess number of deaths (per 1000 deaths) attributable to each 10˚F increase in the same day's summer temperature.

Variable	Excess deaths in 1987	Excess deaths in 2005	Temporal change
**National**	51 (42, 61)*	19 (12, 27)*	-32 (-45, -18)*
**Age**			
Under 65	39 (26, 53)*	26 (13, 39)*	-13 (-36, 10)
65 to 74	48 (31, 64)*	11 (-5, 26)	-37 (-64, -10)*
75 and older	60 (46, 74)*	20 (11, 29)*	-40 (-59, -21)*
**Region**			
Industrial Midwest	50 (31, 68)*	11 (-3, 25)	-39 (-65, -13)*
Northeast (NE)	70 (49, 92)*	26 (10, 43)*	-44 (-74, -14)*
Northwest (NW)	72 (47, 99)*	30 (10, 50)*	-42 (-80, -5)*
Southern California	31 (-2, 65)	46 (20, 72)*	15 (-34, 64)
Southeast (SE)	45 (23, 68)*	10 (-9, 28)	-35 (-70, -1)*
Southwest (SW)	32 (1, 64)*	11 (-13, 35)	-21 (-68, 25)
Upper Midwest	30 (0, 61)*	18 (-6, 43)	-12 (-57, 33)
**Cause of**			
Cardiovascular	55 (42, 68)*	15 (3, 28)*	-40 (-60, -20)*
Respiratory	86 (58, 113)*	1 (-26, 30)	-85 (-132, -37)*
Other	43 (31, 56)*	25 (16, 35)*	-18 (-38, 2)
**Region x age**			
IM x under 65	44 (19, 70)*	9 (-16, 35)	-35 (-78, 8)
IM x 65 to 74	36 (6, 67)*	8 (-24, 40)	-28 (-83, 26)
IM x 75 and older	60 (33, 89)*	12 (-6, 30)	-48 (-86, -11)*
NE x under 65	61 (32, 91)*	14 (-15, 43)	-47 (-98, 3)
NE x 65 to 74	62 (26, 99)*	24 (-13, 62)	-38 (-102, 25)
NE x 75 and older	78 (47, 111)*	31 (10, 52)*	-47 (-91, -4)*
NW x under 65	28 (-10, 68)	58 (20, 98)*	30 (-38, 97)
NW x 65 to 74	86 (39, 136)*	-5 (-49, 41)	-91 (-171, -10)*
NW x 75 and 65	90 (52, 130)*	31 (5, 56)*	-59 (-114, -5)*
older SC x under	16 (-32, 67)	75 (26, 126)*	59 (-29, 146)
SC x 65 to 74	9 (-48, 69)	42 (-16, 104)	33 (-68, 135)
SC x 75 and older	49 (0, 100)	35 (2, 70)*	-14 (-84, 56)
SE x under 65	29 (-5, 65)	29 (-4, 64)	0 (-60, 59)
SE x 65 to 74	59 (17, 103)*	4 (-37, 47)	-55 (-128, 19)
SE x 75 and older	49 (16, 83)*	2 (-22, 27)	-47 (-94, 2)
SW x under 65	27 (-21, 77)	20 (-24, 65)	-7 (-89, 73)
SW x 65 to 74	47 (-9, 108)	-5 (-58, 51)	-52 (-150, 46)
SW x 75 and older	35 (-12, 84)	13 (-18, 46)	-22 (-87, 44)

Variable	Excess deaths in 1987	Excess deaths in 2005	Temporal change
UM x under 65	28 (-18, 76)	23 (-22, 71)	-5 (-85, 77)
UM x 65 to 74	25 (-29, 82)	-2 (-56, 56)	-27 (-121, 67)
UM x 75 and older	29 (-14, 75)	24 (-7, 56)	-5 (-68, 58)

*Denotes statistically significant estimates at the 0.05 level.

Table S2. Effect modification by average temperature over the study period ("Local Climate") and by change in central air conditioning (AC) prevalence over the study period. Posterior mean estimates (95% posterior intervals) of heat-related mortality risk in 1987 and 2005 and of its temporal change from 1987 to 2005 at the 25th and 75th percentiles of each potential effect modifier and of the difference in heat- related mortality risk and in the temporal trends comparing the 25th to 75th percentiles. Heat-related mortality risk is defined as the excess number of deaths (per 1000 deaths) attributable to each 10˚F increase in the same day's summer temperature.

**Variable**	**Excess deaths in 1987**	**Excess deaths in 2005**	**Temporal change**
**Local climate**			
25th percentile (52˚F)	57 (45, 69)*	16 (7, 24)*	-42 (-58, -26)*
75th percentile	42 (29, 56)*	25 (15, 36)*	-17 (-36, 2)
Difference	15 (-1, 31)	-10 (-22, 3)	-25 (-47, -2)*
**Change in central AC**			
25th percentile	57 (45, 69)*	27 (17, 36)*	-31 (-47, -14)*
75th percentile	51 (40, 62)*	15 (6, 24)*	-36 (-52, -20)*
Difference	6 (-8, 20)	12 (1, 23)*	5 (-14, 24)

*Denotes statistically significant estimates at the 0.05 level.

Table S3. Average number of deaths per summer (1987 to 2005) and the excess number of heat-related deaths attributable to a 5˚F increase in average daily temperature.

**City**	**Average deaths per summer (1987 to 2005)**	**Excess attributable to a 5°F shift***
Akron	1028.1	-0.2
Albuquerque	745.2	5.7
Arlington	182.2	2.7
Atlanta	1952.7	12.5
Austin	703.1	5.7
Bakersfield	866.4	12.5
Baltimore	1643.4	40.4
Baton Rouge	622.8	5.1
Biddeford	247.7	1.5
Birmingham	1486.2	9.5
Boston	1063.3	26.1
Buffalo	2187.6	9.9
Cayce	229.1	1.6
Cedar Rapids	272.9	3.7
Charlotte	829.7	5.4
Chicago	9705.9	63.1
Cincinnati	1718.8	4.9
Cleveland	3193.3	43.6
Columbus, GA	342.1	3.2
Columbus, OH	1624.8	-7.4
Colorado Springs	517.7	8.6
Corpus Christi	472.3	6.4
Coventry	258.8	1.6
Dayton	1082.4	22.8
Washington	1253.1	14.4
Des Moines	562.1	1.9
Detroit	3993.1	59.0
Dallas/Fort Worth	4879.3	43.5
El Paso	763.5	6.2
Evansville	376.0	0.7
Fresno	1039.5	12.9
Fort Wayne	517.2	2.1
Grand Rapids	800.9	9.8
Greensboro	642.3	2.8
Houston	3849.2	35.8
Huntsville	385.2	1.3
Indianapolis	1538.5	9.6
Jackson	450.7	3.3

City	Average deaths per summer (1987 to 2005)	Excess attributable to a 5°F shift*
Jacksonville	1252.3	6.6
Jersey City	919.4	15.4
Johnstown	342.1	-0.1
Kansas City, MO	1590.2	17.0
Kansas City, KS	283.1	3.7
Kingston	245.1	0.9
Knoxville	663.7	5.2
Los Angeles	12621.8	250.0
Lafayette	247.2	2.6
Las Vegas	1760.6	6.0
Lexington	374.0	4.5
Lincoln	323.2	1.5
Lake Charles	320.8	1.9
Louisville	1425.4	26.5
Little Rock	662.3	9.4
Lubbock	351.2	0.6
Madison	496.3	4.9
Memphis	1627.8	10.6
Miami	4008.9	52.0
Milwaukee	1882.5	-9.0
Minneapolis/St. Paul	2407.9	29.2
Mobile	739.0	6.3
Modesto	629.8	5.5
Muskegon	310.4	2.9
Nashville	987.5	8.4
Newport News	233.2	2.6
New Orleans	1004.1	11.3
Norfolk	410.3	2.2
Newark	1493.8	25.0
New York	15669.8	258.8
Oakland	1904.2	35.3
Oklahoma City	1222.1	7.0
Olympia	264.7	4.7
Omaha	732.8	6.7
Orlando	1122.1	15.8
Philadelphia	3461.6	60.8
Phoenix	4039.1	22.4
Pittsburgh	3193.6	19.3
Portland	1680.1	4.2
Providence	1242.9	5.7
Raleigh	572.8	2.9
Richmond	398.0	5.3
Riverside	2081.5	39.1

City	Average deaths per summer (1987 to 2005)	Excess attributable to a 5°F shift*
Rochester	1332.9	2.6
Sacramento	1704.0	23.1
Salt Lake City	933.5	1.4
San Antonio	1938.0	35.8
San Bernardino	2017.4	22.3
San Diego	3911.2	58.5
San Francisco	1391.9	21.8
San Jose	1772.9	27.0
Seattle	2319.8	53.4
Shreveport	690.4	8.0
Spokane	726.2	4.6
Santa Ana/Anaheim	3119.4	27.1
St. Louis	806.1	6.8
Stockton	790.6	13.0
St. Petersburg	2634.1	39.0
Syracuse	861.0	1.5
Tacoma	952.4	5.2
Tampa	1646.1	26.8
Toledo	897.3	10.4
Topeka	326.1	2.4
Tucson	1342.6	9.1
Tulsa	976.9	-0.5
Wichita	698.5	3.9
Worcester	1331.5	20.5
Total	166346.7	1907.4

*Calculated by applying a projected 5°F temperature shift to our estimates of the relative risk of mortality associated with a 1°F temperature increase in 2005.
